# Humanistic and socioeconomic burden of COPD patients and their caregivers in Malaysia

**DOI:** 10.1038/s41598-021-01551-5

**Published:** 2021-11-19

**Authors:** Anees ur Rehman, Sohail Ayaz Muhammad, Zermina Tasleem, Alyaa Alsaedi, Mamoona Dar, Muhammad Omer Iqbal, Muhammad Fawad Rasool, Shahid Shah, Ghulam Abbas, Sadia Shakeel, Khezar Hayat

**Affiliations:** 1 Department of Clinical Pharmacy, School of Pharmaceutical Sciences, University Sains Malaysia, Gelugor, Malaysia; 2 Department of Pharmacy Practice, Faculty of Pharmacy, Bahauddin Zakariya University Multan, Multan, Pakistan; 3 School of Management Sciences, University Sains Malaysia, Gelugor, Malaysia; 4 Department of Political Sciences, Bahauddin Zakariya University Multan, Multan, Pakistan; 5 School of Industrial Technology, University Sains Malaysia, Gelugor, Malaysia; 6 College of Pharmacy, University of Sargodha, Sargodha, Pakistan; 7 School of Medicine and Pharmacy, Ocean University of China, Qingdao, China; 8 Faculty of Pharmaceutical Sciences, Government College University, Faisalabad, Pakistan; 9 Department of Pharmacy Practice, Faculty of Pharmaceutical Sciences, Dow University of Health Sciences, Karachi, Pakistan; 10 Institute of Pharmaceutical Sciences, University of Veterinary and Animal Sciences, Lahore, Pakistan

**Keywords:** Health care economics, Quality of life

## Abstract

Chronic obstructive pulmonary disease (COPD) is associated with substantial humanistic and socioeconomic burden on patients and their caregivers. COPD is expected to be 7th leading cause of disease burden till 2030. The objective of the current study was to assess the humanistic and socioeconomic burden of COPD patients and their caregivers in Malaysia. The burden includes the cost of management of COPD, QOL of COPD patients and their caregivers, work productivity and activity impairment of COPD patients and their caregivers due to COPD. One hundred and fifty COPD patients and their caregivers from the chest clinic of Penang Hospital were included in the study from August 2018 to August 2019. Caregiving cost was estimated using the replacement cost approach, while humanistic and social burden was assessed with the help of health status questionnaires. Overall, 64.66% and 7.1% of COPD patients reported to depend on informal caregivers and professional caregivers respectively. COPD patients reported dyspnoea score as 2.31 (1.31), EQ-5D-5L utility index 0.57 (0.23), CCI 2.3 (1.4), SGRQ-C 49.23 (18.61), productivity loss 31.87% and activity impairment 17.42%. Caregivers reported dyspnoea score as 0.72 (0.14), EQ-5D-5L utility index 0.57 (0.23), productivity loss 7.19% and social activity limitation as 21.63% due to taking care of COPD patients. In addition to the huge direct cost of management, COPD is also associated with substantial burden on society in terms of compromised quality of life, reduced efficiency at the workplace, activity impairment and caregiver burden.

## Introduction

Chronic obstructive pulmonary disease (COPD) is associated with substantial humanistic and socioeconomic burden on patients and society^1^. COPD is characterised by persistent airflow limitation. While smoking is the primary risk factor for initiation of COPD, other risk factors include pollution, environmental conditions, occupational exposure, biomass fuel burning, atopy, alpha-1 antitrypsin deficiency, antioxidant deficiency, respiratory infections and asthma^2,3^. It affects more than 10% of the world’s population and is responsible for 3 million deaths every year^4^. Its prevalence is expected to rise in future due to increase in tobacco smoking, urbanisation, industrialisation, exposure to risk factors and aging populations^5^. The Malaysian population is at increased risk of COPD due to the higher prevalence of cigarette smoking (49.2%) among the adult population^6,7^. In 2010, COPD was the 4th leading cause of hospital admissions in Malaysia, causing an economic burden of $673 million on the healthcare system^8^.

Cost estimation from different countries showed that direct cost for the treatment of COPD utilises a significant proportion of the healthcare budget of each country^9–11^. The cost spent by COPD patients was approximately 2.4 times higher than other patients^12^. COPD also results in significant burden to society resulting from productivity losses due to impaired health status, early retirement, and disability pension^13,14^. COPD causes limitations to social behavior and daily activities. It is the 9th foremost cause of disability-adjusted life years (DALY)^15^. It causes a huge burden on the economy of the country due to work productivity loss, early retirement and disability pension. Approximately 40% to 60% of patients diagnosed with COPD are of working age^16^. COPD is the 11th leading cause of disease burden and is expected to be 7th till 2030^17^. Employees diagnosed with COPD experience almost 5-times increase in work productivity loss and 3-times increase in activity limitation as compared to employees without COPD^18^. COPD also reduces the chances of getting employment by 9% compared to normal persons^19^. COPD patients missed an average of 19.4 working days per year due to exacerbation or outpatient visits and 27.5 working days due to productivity loss or compromised performance at work place^20^.

Informal care is an essential part of the care provided to patients suffering from chronic diseases. Informal caregivers (unpaid voluntary family members and friends) assist the patients to perform daily activities, thus improve the quality of life (QOL) of patients by reducing disease burden^21^. In addition to COPD patients, COPD also affects QOL, social activities and the efficiency of caregivers of COPD patients due to added responsibilities of managing patients, thus causing a substantial humanistic burden on society^22^. It affects the ability to perform daily activities and sleeping patterns in more than 50% of COPD patients and their caregivers^23^. In a previous study, 38.9% of caregivers reported reduced working hours, and 11.4% reported quitting their job due to added responsibility of caregiving^22^. Work productivity loss and absenteeism of caregivers due to attending COPD patient effects national economy.

Knowledge about healthcare resource utilisation, associated costs and QOL related to treatment results in optimal use of healthcare resources and cost economical management of disease. To date, clinical trials of new therapies for managing chronic disorders are focusing on cost effective management of COPD in addition to improving clinical measures. Assessment of humanistic and economic burden of COPD can help to understand the long-term clinical, societal, and economic consequences of COPD and can help to plan interventions to reduce the consequences of such burden. Most of the cost related studies have focused on direct and indirect patient related cost^1,9^. Few studies assessed informal care cost (hours spent on caregiving multiplied with hourly wedge) as cost component of total direct cost but studies assessing the complete humanistic burden (QOL, productivity loss and activity impairment) related to caregivers of COPD patients are limited due to complexities associated with collecting data from informal caregivers^24^. Various country guidelines recommend to assess the societal impact of a clinical trial in terms of caregiver burden^25^. Assessment of caregiver burden in economic evaluation can give true picture of the burden of disease. Neglecting caregiver burden (QOL) can result in underestimation of the exact socioeconomic burden of COPD and may result in over or underestimation of the benefits of an intervention^24,26^. Thus, the objective of the current study was to assess the humanistic and socioeconomic burden of COPD patients and their caregivers in Malaysia. The burden includes cost of management of COPD, QOL of COPD patients and their caregivers, work productivity and activity impairment of COPD patients and their caregivers due to COPD.

## Methodology

The study was the part of prospective cohort, held in the chest clinic of Penang Hospital during August 2018 to August 2019. Purposeful sampling was done to include patients with caregivers. 150 patients and their caregivers were included in the study. The inclusion criterion was (i) patients with confirmed diagnosis of COPD according to GOLD guidelines, (ii) post bronchodilator forced expiratory volume in 1 s/forced vital capacity (FEV1/FVC) ratio < 70%), (iii) minimum of 6-months outpatient record to avoid abrupt changes in QOL due to initiation of therapy, (iv) no changes in treatment over the past 4 weeks, (v) no other respiratory disorders, (vi) no other diseases that have a short-term effect on QOL, and^6^ no disability. This study was conducted in accordance with the Declaration of Helsinki.

### Data collection

**Figure 1:**
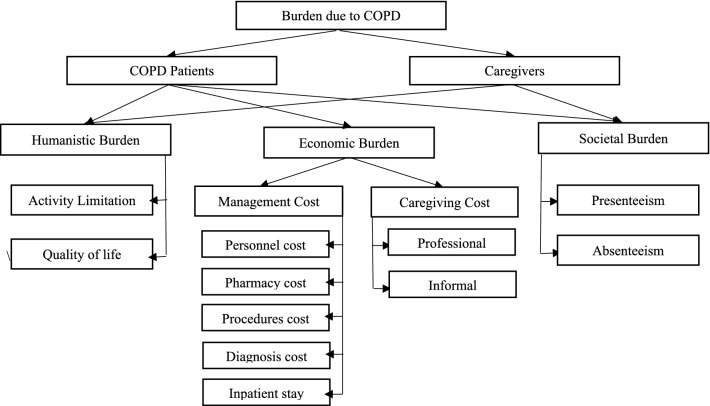
Humanistic, economic and societal burden due to COPD on patients and their caregivers.

A self-administered questionnaire was used to collect information about demographics, social and employment status, smoking status, clinical measures, exacerbation frequency, economics data, dyspnoea, comorbidities (based on Charlson comorbidity index), indirect cost, exercise capacity and QOL. Components of humanistic, economic and societal burden due to COPD on patients and their caregivers is shown in Fig. 1.

Questionnaire was completed by the patients and their attendants during waiting time before consultation. Help was provided by non-technical staff, if someone was unable to complete the questionnaire due to poor eyesight, unable to read or shaky hands.

#### Direct cost

Direct cost for the management of COPD was derived from our previously published cost study on economic burden of COPD in Malaysia^14^. In brief a combination of top-down approach and bottom-up approach was used to assess the direct cost of management of COPD (maintenance phase and exacerbation phase). Economic data was obtained from patients and officials of hospital administrative and finance departments. Different cost centres identified in management of COPD were chest clinic, spirometry room, pathology and radiology laboratories, outpatient and inpatient pharmacy, emergency department (ED), and respiratory ward. All the activities in cost centres were observed prospectively to evaluate the direct cost. Costs from these cost centres and the unit prices for the services provided were calculated and incurred directly to the patients receiving the services. Malaysian official cost tariffs were used for cost calculation^27^. All the costs were calculated in Malaysian ringgit and converted to US$ 2019 rates (1US$ = 4.13MYR on September 18, 2019)^28^.

#### Caregivers cost

Caregiving cost was estimated using replacement cost approach, which values caregiver time at the wage rate or market price of a closest substitute. For professional caregiver average monthly wage of a nurse was used. Whereas, for informal caregivers we used an average monthly wage of unskilled Malaysian population because most informal caregivers are family members, not professionally trained health workers. Caregiver time was reported by patients and further verified by the caregivers^29^. The recall period was one week for house hold activities and 3 months for medical visits. Data related to personal care, household assistance, and practical support during hospital visits were collected. Daily care hours were multiplied by 365 to get annual informal care hours. Annual informal care giving hours were multiplied with the average hourly wage of the unskilled Malaysian population. Whereas, annual professional home care giving hours were multiplied with the average hourly wage of a nurse.

#### Indirect costs

The indirect cost due to work productivity and activity impairment was calculated for working patients and their caregivers. Most of the caregivers were informal caregivers. The work Productivity and Activity Impairment Questionnaire: General Health V2.0 (WPAI-GH) was used to assess the impact of COPD on work productivity loss and activity limitations.

WPAI-GH measures the amount of absenteeism (work time lost due to disease), presenteeism (reduced efficiency due to disease while at work), and activity impairment (restriction in daily activities). For caregivers “activity impairment” was replaced with “social activity limitation”. Recall period for this questionnaire was 7 days. Average missing hours per week were calculated and multiplied with 52 to get the annual missing hours. Absenteeism % was calculated by dividing missing hours with total working hours. Average per person monthly salary was obtained from the Stats department of Malaysian government official website^27^. The latest stats were available for 2017. Unit costs per hour was calculated by dividing their yearly salary by the number of productive working hours per year. Productive working hours were based on average number of shifts per month excluding annual leaves and sick leaves.

#### Clinical measures

Spirometry was performed according to American thoracic society guidelines^30^. Spirometry was performed 45 min after bronchodilation with 400 μg salbutamol via a spacer. FEV_1_% predicted values were calculated based on reference values from the GOLD guidelines^31^.

Six-minute walk distance test (6MWT) to assess the exercise capacity was conducted according to ATS guidelines in long hospital corridor adjacent to the chest clinic. The distance covered by the patients in six minutes was recorded in meters^32^.

#### Health status measures

Modified medical research council (mMRC) dyspnoea scale was used to assess the degree of breathlessness in COPD patients. The mMRC assess the level of breathlessness and its impact on daily activities on a scale from 0 to 4, with 4 representing the worst dyspnoea^33^.

Comorbidity burden was estimated with Charlson comorbidity index (CCI). The CCI evaluates the comorbidity burden by weighing and summing the patient reported conditions (e.g. dementia, peptic ulcer, myocardial infarction, liver disease, congestive heart failure, diabetes mellitus, peripheral vascular disease, connective tissue disease, chronic kidney disease, hemiplegia, cancer, lymphoma, leukaemia, cerebrovascular accident and AIDS). Higher score indicates greater comorbidity burden^34^.

Malaysian version of St. George’s Respiratory COPD specific questionnaire (SGRQ-C) was used to assess the QOL of patients^35^. SGRQ-C is a self-administered health status measure used to assess the impact of disease on psychological and social functioning of COPD patients. The questionnaire consists of symptoms, activity and impact subscale. The symptom subscale collects information about respiratory symptoms (cough, sputum, wheeze and dyspnoea), activity subscale collects information about limitation of activities of COPD and impact subscale collects information about the impact of activity limitation on person and society due to COPD. Each subscale score and total score range from 0 to 100, with 100 shows the worst QOL^36^.

European quality of life 5-Dimension 5-Level questionnaire (EQ-5D-5L) was used to assess the QOL of COPD patients and their caregivers^37^. EQ-5D-5L is a generic QOL instrument. It is widely used in economic evaluations worldwide. The utility index (EQ-UI) is calculated from the descriptive scale of five components (mobility, self-care, usual activities, pain and depression). Patients mark each dimension on a scale of 1 “no problem” to 5 “worst problem”. The five-digit number (ranging from 11,111 to 55,555) obtained was then converted to a utility index based on EQ-5D-5L value set for Malaysia^38^. EQ-5D-5L also includes a vertical visual analogue scale (EQ-VAS) to measure general QOL from 0 (worst possible health) to 100 (best possible health). Higher EQ-5D-5L UI and EQ-VAS values reflect good health status. It is easy to use and interpret and allows the comparison of QOL of diseased persons with healthy population.

### Statistical analysis

Frequencies and percentages were reported for categorical variables and the mean and standard deviation were reported for continuous variables. Normality of the data was checked using Shapiro–Wilk test. Chi-square tests was used to compare categorical variables and independent-samples t-test was used to compare continuous variables among COPD patients and their caregivers. A p value of < 0.01 was considered to be statistically significant. Statistical analyses were performed using IBM SPSS Statistics V24.0 (IBM Corporation, Armonk, NY, USA).

### Ethics approval and consent to participate

Ethical approval for this study was obtained from the Medical Research and Ethics Committee (MREC), Ministry of Health Malaysia (Registration number: NMRR-18-1482-42075). Written informed consent was obtained from all participants.

### Consent for publication

We would like to thank the Director-General of Health, Malaysia for his permission to publish this study.

## Results

One hundred and fifty COPD patients and one hundred twenty-seven caregivers were included in the disease burden analysis. Among the included patients 104 (69.33%) were male, with BMI 24.14 (4.67) kg/m^2^, FEV1% predicted 53.74% (12.84) and 6MWT as 421.5 (65.28) meters. Whereas, 48 (37.79%) caregivers were male with mean age 41.28 (4.18) years and BMI 20.97 (3.19) kg/m2. Mean CCI score was reported as 2.31 (1.31) in COPD patients, whereas mean CCI in caregivers was reported as 0.52 (0.35). MMRC dyspnoea score was reported as 2.31 (1.31) and 0.72 (0.14) in COPD patients and their caregivers respectively. Significant difference was observed in age, BMI, smoking status, mMRC dyspnoea score and CCI score among COPD patients and their caregivers. Socio-demographic and clinical characteristics of the COPD patients and their caregivers are displayed in Table 1.

**Table 1:** Sociodemographic and clinical characteristics of the COPD patients and their caregivers.

	COPD patients	COPD caregivers	p value
No. of patients (%)	150	127	–
BMI	24.14 (4.67) kg/m^2^	20.97 (3.19) kg/m^2^	< 0.01
Male	104 (69.33%)	48 (37.79%)	0.12
Mean age	61.56 (8.3)	49.38 (8.5)	< 0.01
**Ethnicity**			
Malay	37 (24.66%)	32 (25.91%)	0.54
Chinese	65 (43.33%)	62 (48.81%)	0.67
Tamil	44 (29.33%)	31 (24.40%)	0.34
Others	4 (2.66%)	2 (2.06%)	0.09
**Working status**			
Retired	29 (19.33%)	14 (14.43%)	0.25
Full time	96 (64.0%)	95 (74.80%)	0.12
Part-time	25 (16.67%)	18 (14.17%)	0.67
**Smoking status**			
Current smokers	19 (12.66%)	49 (38.58%)	< 0.01
Ex-smoker	107 (71.33%)	18 (14.17%)	< 0.01
Never smoker	24 (16%)	60 (47.24%)	< 0.01
Years with COPD	6.7 (5.23)	–	
**Charlson comorbidity index**			
CCI	2.3 (1.4)	0.52 (0.35)	< 0.001
**Post-bronchodilator spirometry**			
FEV_1_%	53.74 (12.84)		
FEV1/FVC	51.36 (11.14)		
**Medication**			
LABA	103 (68.66%)		
LAMA	94 (62.66%)		
ICS	47 (31.33%)		
**Exercise capacity**			
6MWT in meters	421.5 (65.28)		
**Percentage of patients according to severity of COPD**			
GOLD grade I	25 (16.60)		
GOLD grade II	44 (29.33)		
GOLD grade III	53 (35.33		
GOLD grade IV	28 (18.67)		
**Exacerbations**			
Hospitalization	46 (30.67%)		
Exacerbation per year	1.27 (1.4)		
Hospital stay days	4.9 (4.8)		

Results were displayed as mean (standard deviation) and number (percentage) unless otherwise stated.

BMI, Body mass index in kg/m^2^; COPD, Chronic obstructive pulmonary disease; CCI, Charlson comorbidity index; FEV^1^, forced expiratory volume in one second; FVC, forced vital capacity; FEV1%, % predicted FEV_1_; GOLD 1, FEV_1_ ≥ 80% predicted; GOLD II, FEV_1_ 50% to 80% predicted; GOLD III, FEV_1_ 30% to 50% predicted; GOLD IV, FEV_1_ < 30% predicted; ICS; Inhaled Corticosteroids; LABA, long-acting beta agonist; LAMA, long acting muscarinic antagonist.

### Direct cost for management of COPD:

Mean annual per-patient direct cost for the treatment of COPD was calculated as US$586.78. In detail pharmacy cost was highest as US$370.80, followed by inpatient stay cost US$ 103.09, personnel Cost US$52.77, others (cleaning, electricity, communication, building maintenance, and depreciation cost) US$16.42, pathological laboratory tests cost US$14.18, procedures (ventilation) cost US$13.30, X-ray cost US$6.80 and Spirometry cost US$4.58. The mean annual cost of transportation per COPD patient to attend outpatient visits, emergency department visits and hospitalisation was calculated as $8.57. Socioeconomic burden of COPD patients and their caregivers is displayed in Table 2.

**Table 2:** Societal and economic burden of COPD patients and their caregivers.

**Direct cost**	
Personnel cost	$52.77 (24.49)
Pharmacy cost	$370.80 (216.5)
Procedure’s cost	$13.30 (7.4)
X-ray cost	$6.80 (2.05)
Spirometry cost	$4.58 (2.61)
Laboratory cost	$14.18 (3.03)
Inpatient cost	$103.09 (58.59)
Others^a^	$16.42 (10.34)
Direct non-medical cost	$8.57 (3.71)
Total direct cost	US$586.78
**Caregiving cost**	
Informal caregiver cost^b^	$347.63 (157.38)
Professional caregiver cost^c^	$1001.96 (337.42)
Total caregiving cost	US$1349.59
**Indirect cost**	
COPD patients	$1699.76 (627.26)
Caregivers	$217.92(87.72)
Total indirect cost	US$1917.68

All costs in US$ (United States dollar); 1US$ = 4.13MYR on September 18, 2019; mean (standard deviation); COPD, Chronic obstructive pulmonary disease.

^a^Others, (cleaning, electricity, communication, building maintenance, and depreciation cost).

^b^64.66% of COPD patients were depending on informal caregivers.

^c^7.1% of COPD patients utilised professional healthcare services at home.

### Informal caregiver cost

Overall, 64.66% of COPD patients were depending on caregivers to perform daily activities. Among these patients mean annual caregiver hours were reported as 195.6 h. Mean annual informal caregiver cost was calculated as $347.63. Among the patients relying on informal care 48% reported to have one informal caregiver, 39% reported to have 2 informal caregivers and 13% reported to have 3 or more informal caregivers.

A total of 7.1% of COPD patients reported using professional healthcare services at home. The average salary of the nurse was reported as $468.77 per month. On average a patient received 342 h of professional healthcare services in home per year. Mean annual health professional services cost at home was calculated as $1001.96 per patient.

### Indirect cost of COPD patients and their caregivers

Only those patients and their caregivers who were currently employed were included in indirect cost analysis. Approximately 80.66% of COPD patients and 81.44% of caregivers were currently employed. The mean annual indirect cost per COPD patient was calculated as $1699.76 due to COPD and $377.72 due to other reasons. Productivity loss at working place and activity limitations were reported as 31. 87% and 17.42% respectively.

In addition to assisting in daily activities, caregivers also accompany patients during outpatient visits and hospitalisation. Mean annual indirect cost of the caregivers was calculated as $217.92. Productivity loss due to taking care of COPD patients was calculated as 7.19% and social activity limitation was reported as 21.63%. Mean indirect cost of patients and their attendants are displayed in Table 3.

**Table 3:** Indirect cost of patients and their attendants.

	COPD patients	COPD caregivers	p value
Currently employed	12 (80.67%)	113 (88.97%)	0.42
Absenteeism %^a^	26.37% (7.12)	4.17%	< 0.01
Absenteeism due to other reasons	5.46% (1.86)	11.34%(4.28)	< 0.01
Presenteeism %	31.87% (9.98)	7.19% (2.1)	< 0.01
Activity impairment %^b^	27.42% (6.59)	21.63% (8.5)	0.17

All results displayed as mean (standard deviation) unless otherwise stated; absenteeism, work time lost due to COPD or taking care of COPD patient; activity impairment, restriction in daily activities; presenteeism, reduced productivity while at work due to COPD or taking care of COPD patients.

^a^Absenteeism % was calculated by dividing missing hours with total working hours.

^b^For caregivers activity limitation was considered as social activity limitation due to taking care of COPD patient.

Significant difference was observed in missing hours from work due to COPD and productivity loss on working place among COPD patients and their caregivers.

### Quality of life of COPD patients and their caregivers

31.49% of caregivers and 14% patients reported best health status according to EQ-5D-5L. Significant differences were observed in health status of COPD patients and their caregivers. Humanistic burden of COPD on patients and their caregivers is displayed in Table 4.

**Table 4:** Humanistic burden due to COPD on patients and their caregivers.

	COPD patients	Caregivers	p value
**Dyspnoea**			
mMRC dysponea	2.31 (1.31)	0.72 (0.14)	< 0.01
**EQ-5D-5L**			
EQ-5D-5L UI	0.57 (0.23)	0.76 (0.36)	< 0.01
EQ-VAS	53.24 (17.30)	85.64 (14.52)	< 0.01
**SGRQ-C**			
SGRQ-C symptom score	53.44 (19.98)		
SGRQ-C activity score	48.43 (18.78)		
SGRQ-C impact score	43.37 (20.71)		
SGRQ-C total	49.23 (18.61)		

All results displayed as mean (standard deviation) unless otherwise stated.

EQ-5D-5L, European quality of life 5-Dimension 5-Level questionnaire; EQ-UI, EQ-5D-5L utility index; EQ-VAS, EQ-5D-5L vertical visual analogue scale; mMRC dyspnoea, Modified medical research council dyspnoea scale; SGRQ-C, Malaysian version of St. George’s Respiratory COPD specific questionnaire.

## Discussion

Results of the study endorse that COPD is associated with substantial clinical, humanistic, societal and economic burden on patients and their caregivers. COPD patients depend on their family members to perform daily activities. In working age patients and caregivers, COPD affects the financial life by causing days away from work and inability to perform with full efficiency on work place^39^. Among the study participants, majority of the COPD patients and caregivers were currently employed. COPD patients and caregivers were associated with substantial productivity loss in work life and activity impairment. Our results are in line with the study of Mazanec et al. who reported that caregivers can’t work with full efficiency on work place and delay tasks due to reduced personal rest time^37^. Caregivers miss working hours and compromise productivity due to travel for treatment, accompanying patient during outpatient visit or hospital admission and providing care in home^40^. Policies like an innovative healthcare service offering, for example, patient transport service to the site of treatment and better nursing care during hospital admission can reduce the burden on caregivers. In addition caregiver education programs, and informal support from family members to reduce care time and increase of caregivers’ free time can also reduce caregiver burden^41^. A recent study from Spain reported that, approximately 20% reduction in productivity loss could result in annual savings of $3.43 billion^42^.

COPD is a multifactorial health problem, having substantial impact on QOL of patients and their caregivers^43^. Humanistic burden due to COPD on patients and caregivers was assessed with SGRQ-C, EQ-5D-5L and mMRC dyspnoea scale. COPD Patients and their caregivers showed compromised QOL. In patients, COPD may cause complete restriction of activities, or slowing down the activities^44^. The possible reason for impaired QOL in COPD patients can be the progressive nature of disease. Due to continuous progression of disease patients focus more on worsening of activities than improvement. Caregivers may also have impaired quality of life due to added responsibilities and unwanted tasks. A previous study reported that more than 50% of caregivers were suffering from depression due to health status of their partners^45^. Patient reported outcomes i.e. degree of dyspnoea, exercise capacity, activity limitation and health related QOL are reliable predictors of various disease markers including wellbeing, severity and mortality^44^. Self-management programs, pulmonary rehabilitation programs, and workplace adaptations can help to improving the QOL of the patients and their caregivers^3^.

This is the single centered study which may affect the generalisation of our results. However, we used tariff-based cost estimates which are applicable across Malaysia. This may result in improving the generalizability of our results. Despite few limitations the study has several strengths. This study’s findings will provide the most up-to-date data on the disease burden associated with COPD, which can be helpful in the planning of healthcare needs and allocation of optimal resources. Moreover, data published on caregiver cost of COPD are rare. For caregiver cost, data was collected directly from the caregivers and cross-checked with the patients, which give more reliable results. Many national and international Pharmacoeconomic guidelines recommend considering the impact of the societal benefit of an intervention before considering it cost-effective especially for chronic disease like COPD which possess a substantial burden on patient and society. So, including caregiver cost can provide an opportunity for the healthcare professionals and researchers to compare the social welfare impact of future interventions in cost-effectiveness studies related to COPD.

## Conclusions

This study provides the most up-to-date information on the humanistic, economic and societal burden associated with COPD. Results of the study endorse that in addition to huge direct cost of management, COPD is also associated with substantial burden on society in terms of compromised, quality of life, reduced efficiency at workplace, activity impairment and caregiver burden. Assessment of true complete burden of COPD can be helpful in planning of healthcare needs and allocation of optimal resources. Inclusion of caregiver burden in economic evaluation studies can represent the societal burden associated with disease. So, including caregiver cost can provide an opportunity for researchers to compare the social welfare impact of future interventions in cost-effectiveness studies related to COPD.

## Data Availability

Data is available on request from corresponding author.

## References

[1] ur Rehman A, Ahmad Hassali MA, Muhammad SA, Shah S, Abbas S, Hyder Ali IAB (2020). The economic burden of chronic obstructive pulmonary disease (COPD) in the USA, Europe, and Asia: Results from a systematic review of the literature. Expert Rev. Pharmacoecon. Outcomes Res..

[2] GOLD. Global initiative for chronic obstructive lung disease (2019) https://goldcopd.org/wp-content/uploads/2018/11/GOLD-2019-v1.6-FINAL-08Nov2018-wms.pdf. Accessed 01 September 2019.

[3] ur Rehman, A. *et al.* Pharmacological and non-pharmacological management of COPD; limitations and future prospects: A review of current literature. *J. Public. Health* **28**(4), 357–366, (2020).

[4] López-Campos JL, Tan W, Soriano JB (2016). Global burden of COPD. Respirology.

[5] Rehman AU, Shah S, Abbas G, Harun SN, Shakeel S, Hussain R (2021). Assessment of risk factors responsible for rapid deterioration of lung function over a period of one year in patients with chronic obstructive pulmonary disease. Sci. Rep..

[6] Board MHP. ITC Malaysia National Report: findings from Wave 1 to 4 surveys (2005–2009) (2012) http://www.mysihat.gov.my/v2/promosi/images/stories/pusatmaklumat/itcreport. Accessed 01 September 2019.

[7] Shahab L, Jarvis M, Britton J, West R (2006). Prevalence, diagnosis and relation to tobacco dependence of chronic obstructive pulmonary disease in a nationally representative population sample. Thorax.

[8] Hassan HA, Aziz NA, Hassan Y, Hassan F (2014). Does the duration of smoking cessation have an impact on hospital admission and health-related quality of life amongst COPD patients?. Int. J. Chron. Obstruct. Pulmon. Dis..

[9] ur Rehman A, Hassali MAA, Muhammad SA, Harun SN, Shah S, Abbas S (2020). The economic burden of chronic obstructive pulmonary disease (COPD) in Europe: Results from a systematic review of the literature. Eur. J. Health Econ..

[10] Shah S, Abbas G, Hanif M, Anees-Ur-Rehman, Zaman M, Riaz N (2019). Increased burden of disease and role of health economics: Asia-pacific region. Expert Rev. Pharmacoecon. Outcomes Res..

[11] Shah S, Abbas G, Riaz N, ur Rehman A, Hanif M, Rasool MF (2020). Burden of communicable diseases and cost of illness: Asia pacific region. Expert Rev. Pharmacoecon. Outcomes Res..

[12] de Oca MM, Aguirre C, Varela MVL, Laucho-Contreras ME, Casas A, Surmont F (2016). exacerbations and health care resource utilization in patients with airflow limitation diseases attending a primary care setting: The PUMa study. Int. J. Chron. Obstruct. Pulmon. Dis..

[13] Ding D, Kolbe-Alexander T, Nguyen B, Katzmarzyk PT, Pratt M, Lawson KD (2017). The economic burden of physical inactivity: A systematic review and critical appraisal. Br. J. Sports Med..

[14] Ur Rehman A, Hassali MAA, Muhammad SA, Shakeel S, Chin OS, Ali IA (2021). Economic burden of chronic obstructive pulmonary disease patients in Malaysia: A longitudinal study. PharmacoEcon.-open.

[15] Soriano JB, Abajobir AA, Abate KH, Abera SF, Agrawal A, Ahmed MB (2017). Global, regional, and national deaths, prevalence, disability-adjusted life years, and years lived with disability for chronic obstructive pulmonary disease and asthma, 1990–2015: A systematic analysis for the Global Burden of Disease Study 2015. Lancet Respir. Med..

[16] Rai KK, Adab P, Ayres JG, Siebert WS, Sadhra SS, Sitch AJ (2017). Factors associated with work productivity among people with COPD: Birmingham COPD Cohort. Occup. Environ. Med..

[17] WHO. Measurement of Healthy Life Expectancy and Wellbeing. (World Health Organization) https://www.who.int/healthinfo/sage/meeting_reports/en/.

[18] daCosta DM, Paulose-Ram R, Su J, McDonald M, Zou KH, Wagner J-S (2012). The burden of chronic obstructive pulmonary disease among employed adults. Int. J. Chron. Obstruct. Pulmon. Dis..

[19] Thornton Snider J, Romley JA, Wong KS, Zhang J, Eber M, Goldman DP (2012). The disability burden of COPD. COPD J. Chron. Obstruct. Pulm. Dis..

[20] May, S. M. & Li, J. T. (eds). Burden of chronic obstructive pulmonary disease: healthcare costs and beyond. In *Allergy and Asthma Proceedings* (OceanSide Publications, 2015). 10.2500/aap.2015.36.3812 PMC5554331 25562549

[21] Martire LM, Lustig AP, Schulz R, Miller GE, Helgeson VS (2004). Is it beneficial to involve a family member? A meta-analysis of psychosocial interventions for chronic illness. Health Psychol..

[22] Erdal M, Johannessen A, Askildsen JE, Eagan T, Gulsvik A, Grønseth R (2014). Productivity losses in chronic obstructive pulmonary disease: A population-based survey. BMJ Open Respir. Res..

[23] Guarascio AJ, Ray SM, Finch CK, Self TH (2013). The clinical and economic burden of chronic obstructive pulmonary disease in the USA. ClinicoEcon. Outcomes Res..

[24] Krol M, Papenburg J, van Exel J (2015). Does including informal care in economic evaluations matter? A systematic review of inclusion and impact of informal care in cost-effectiveness studies. Pharmacoeconomics.

[25] House W, Team CP (2010). Recognised, Valued and Supported: Next Steps for the Carers Strategy.

[26] Goodrich K, Kaambwa B, Al-Janabi H (2012). The inclusion of informal care in applied economic evaluation: A review. Value Health..

[27] Department of Statistics Malaysia op. Household Income & Expenditure (2019) https://www.dosm.gov.my/v1/index.php?r=column/ctwoByCat&parent_id=119&menu_id=amVoWU54UTl0a21NWmdhMjFMMWcyZz09. Accessed 01 September 2019.

[28] Exchange Rates, Central Bank of Malaysia. (Government of Malaysia, 2019) https://www.bnm.gov.my/index.php?ch=statistic&pg=stats_exchangerates. Accessed 01 September 2019.

[29] Dixon S, Walker M, Salek S (2006). Incorporating carer effects into economic evaluation. Pharmacoeconomics.

[30] Association MSotAL. *American Thoracic Society Standardization of Spirometry, 1994 Update* (2012).

[31] Quanjer, P. H. *et al.* Multi-ethnic reference values for spirometry for the 3–95-yr age range: The global lung function 2012 equations. *Eur. Respir. Soc.* **40**(6),1324–43 (2012). 10.1183/09031936.00080312 PMC3786581 22743675

[32] Laboratories ACoPSfCPF (2002). ATS statement: Guidelines for the six-minute walk test. Am. J. Respir. Crit. Care Med..

[33] Mahler DA. mMRC (Modified Medical Research Council) Dyspnea Scale https://www.mdcalc.com/mmrc-modified-medical-research-council-dyspnea-scale#creator-insights. Accessed 01 September 2019.

[34] Charlson ME, Pompei P, Ales KL, MacKenzie CR (1987). A new method of classifying prognostic comorbidity in longitudinal studies: Development and validation. J. Chron. Dis..

[35] Rehman AU, Hassali MAA, Harun SN, Abbas S, Muneswarao J, Ali IABH (2020). Validation and clinical interpretation of the St George’s respiratory questionnaire for COPD (SGRQ-C) after adaptation to Malaysian language and culture, in patients with COPD. Health Qual. Life outcomes.

[36] Meguro M, Barley EA, Spencer S, Jones PW (2007). Development and validation of an improved, COPD-specific version of the St. George Respiratory Questionnaire. Chest.

[37] Mazanec SR, Daly BJ, Douglas SL, Lipson AR (2011). Work productivity and health of informal caregivers of persons with advanced cancer. Res. Nurs. Health.

[38] Shafie AA, Thakumar AV, Lim CJ, Luo N, Rand-Hendriksen K, Yusof FAM (2019). EQ-5D-5L valuation for the Malaysian population. Pharmacoeconomics.

[39] Chapman K, Mannino D, Soriano J, Vermeire P, Buist AS, Thun M (2006). Epidemiology and costs of chronic obstructive pulmonary disease. Eur. Respir. J..

[40] Yamauchi H, Nakagawa C, Fukuda T (2017). Social impacts of the work loss in cancer survivors. Breast Cancer.

[41] ur Rehman A (2019). Utilization of short message service (SMS) in non-pharmacological management of hypertension. A pilot study in an URBAN public hospital of Multan, Pakistan.. Journal of Public Health.

[42] Colás C, Brosa M, Antón E, Montoro J, Navarro A, Dordal M (2017). Estimate of the total costs of allergic rhinitis in specialized care based on real-world data: The FERIN Study. Allergy.

[43] Wang DY, Ghoshal AG, Muttalif ARBA, Lin H-C, Thanaviratananich S, Bagga S (2016). Quality of life and economic burden of respiratory disease in Asia-Pacific—Asia-pacific burden of respiratory diseases study. Value Health Regional Issues..

[44] Rasool MF, Rehman Au, Imran I, Abbas S, Shah S, Abbas G (2020). Risk Factors Associated With Medication Errors Among Patients Suffering From Chronic Disorders.. Front Public Health.

[45] Ohno S, Chen Y, Sakamaki H, Matsumaru N, Tsukamoto K (2020). Humanistic and economic burden among caregivers of patients with cancer in Japan. J. Med. Econ..

